# Exercise for Postmenopausal Bone Health – Can We Raise the Bar?

**DOI:** 10.1007/s11914-025-00912-7

**Published:** 2025-04-10

**Authors:** Shejil Kumar, Cassandra Smith, Roderick J. Clifton-Bligh, Belinda R. Beck, Christian M. Girgis

**Affiliations:** 1 Endocrinology Department, Royal North Shore Hospital, Sydney, Australia; 2 Endocrinology Department, Westmead Hospital, Sydney, Australia; 3 Faculty of Medicine & Health, University of Sydney, Sydney, Australia; 4 School of Medical and Health Sciences, Nutrition & Health Innovation Research Institute, Edith Cowan University, Perth, Australia; 5 Medical School, The University of Western Australia, Perth, Australia; 6 Kolling Institute of Medical Research, Royal North Shore Hospital, Sydney, Australia; 7 School of Health Sciences & Social Work, Griffith University, Gold Coast Campus, Australia

**Keywords:** Exercise, Resistance exercise, Weightbearing exercise, Osteoporosis, Osteopenia, Postmenopausal, Fragility fractures

## Abstract

**Purpose of Review:**

This review summarises the latest evidence on effects of exercise on falls prevention, bone mineral density (BMD) and fragility fracture risk in postmenopausal women, explores hypotheses underpinning exercise-mediated effects on BMD and sheds light on innovative concepts to better understand and harness the skeletal benefits of exercise.

**Recent Findings:**

Multimodal exercise programs incorporating challenging balance exercises can prevent falls. Emerging clinical trial evidence indicates supervised progressive high-intensity resistance and impact training (HiRIT) is efficacious in increasing lumbar spine BMD and is safe and well-tolerated in postmenopausal women with osteoporosis/osteopenia. There remains uncertainty regarding durability of this load-induced osteogenic response and safety in patients with recent fractures. Muscle-derived myokines and small circulating extracellular vesicles have emerged as potential sources of exercise-induced muscle-bone crosstalk but require validation in postmenopausal women.

**Summary:**

Exercise has the potential for multi-modal skeletal benefits with i) HiRIT to build bone, and ii) challenging balance exercises to prevent falls, and ultimately fractures. The therapeutic effect of such exercise in combination with osteoporosis pharmacotherapy should be considered in future trials.

## Introduction

Osteoporosis and fragility fractures constitute a major public health burden, and its prevalence continues to rise in our ageing population, with postmenopausal women being at the highest risk [1, 2]. Over the past three decades, several pharmacotherapeutic approaches (antiresorptive agents e.g. bisphosphonates, denosumab and osteoanabolic agents e.g. romosozumab, teriparatide) have been developed and proven to reduce fracture risk in postmenopausal osteoporosis [3]. Despite this, the incidence of fragility fractures is still rising, and lifetime prevalence remains very high [1, 2]. In developed countries, one-third of women over the age of 50 years will experience an osteoporotic fracture during their remaining lifetime, commonly associated with devastating consequences such as pain and worse quality of life, mobility, independence and survival [1, 4–7].

Exercise, has the potential to target fracture risk reduction through numerous avenues, including i) improvements in balance and falls risk reduction, ii) maintenance or gains in bone mineral density (BMD) and iii) beneficial changes in bone structure. There is emerging clinical trial evidence that high-intensity resistance and impact training (HiRIT) is more beneficial to lumbar spine BMD than traditionally prescribed low to moderate-intensity regimens [8]. Further, HiRIT can be performed safely by postmenopausal women with osteoporosis or osteopenia at elevated risk of fracture, when ensuring certain safety principles are in place, including supervision, individualisation and progressive intensity increments [4]. Overall, these skeletal benefits of exercise may translate to fracture-risk reduction given i) majority of fragility fractures, particularly hip fractures, occur secondary to a fall [1, 9] and ii) given BMD improvement is a surrogate marker for fracture risk reduction [10].

In this review, we summarise the latest evidence on skeletal benefits of exercise in postmenopausal women (falls prevention, BMD, fracture-risk reduction), discuss potential mechanisms driving the relationship between skeletal loading and increased BMD and critically discuss future directions in the field, including whether multi-modal combinations between exercise and osteoporosis medication should be further explored. Comprehensive reviews on the effects of exercise on bone health throughout the lifespan [11], in older men [12] and in individuals with sarcopenia [13] and frailty [14] can be found elsewhere.

### Evidence for Exercise as a Falls Prevention Strategy in Postmenopausal Women

The majority (> 90%) of fragility hip fractures occur secondarily to falls [1, 9]. Hence, exercise interventions which can reduce the risk of falls in postmenopausal women may impart meaningful benefit on falls-related fractures. A recent Cochrane review of 116 randomised controlled trials (RCTs) in healthy adults ≥ 60 years (n = 25,160, 74% female) found an overall 23% reduction in rate of falls with exercise interventions compared to usual care or control exercise (rate ratio 0.77, 95% CI 0.71–0.83, high-certainty evidence) [15]. A ~ 20–30% reduction in fall rates and risk of falling have consistently been reported in other meta-analyses of exercise RCTs in community-dwelling older adults [16, 17]. Programs that involve balance and functional exercises, or are multi-component in nature (balance, functional and resistance exercises) are more beneficial for falls prevention than any individual exercise component [15–17]. A total weekly dose of at least 1.5–3.0 h of multimodal exercise for a minimum of three to six months is required to observe an effect however the most effective exercise prescription for falls prevention is not established due to lack of head-to-head trials [15, 17, 18]. Crucial elements of falls prevention programs are incorporation of exercises that challenge balance [17] and simulate real-life scenarios which carry particular risk for falling. Limitations of these meta-analyses include substantial heterogeneity, unclear risk of bias, poor adherence to exercise and ascertainment bias (with retrospective recall of falls history) [16, 17].

Finnegan et al. assessed long-term persistence of falls reduction by examining trials in community-dwelling older adults (> 65 years) who underwent predominantly gait, balance and functional training with greater than 12-months follow-up [19]. The few trials with over 24-months follow-up achieved a 17% lower risk of falling but no sustained effect on falls reduction. This apparent diminishing effect on falls reduction with longer follow-up may relate to attrition affecting statistical power, limited adherence/persistence to exercise, or a waning of effect over time [19]. It is unclear whether supervision mediates efficacy and safety during falls prevention exercise programs, as this detail is often not reported in trials [17]. However, Sherrington et al. suggested no additional benefit with group exercise and that delivery by a healthcare professional was not required to achieve a reduction in falls risk [15]. In a meta-analysis, telehealth-based exercise interventions in older adults (combination of strength and balance, low-to-moderate intensity, median three sessions/week for 4-months) to improve physical function appeared to be well-tolerated with reasonable adherence but improvements in mobility and balance were modest with insufficient data on falls prevention [20]. Telehealth-based exercise interventions require further investigation and may have a crucial role in optimising engagement with falls prevention strategies in postmenopausal women in the workforce or frail individuals.

### Effects of Exercise on Bone Mineral Density in Postmenopausal Women

#### High-intensity Resistance and Impact Training (HiRIT) is the Most Efficacious Exercise for Improving Bone Mass

According to the principles of *osteogenic loading* derived from animal studies, applying higher strains at a rapid frequency in a weightbearing position provides the most robust stimulus for load-induced adaptations in bone strength [21]. Recently, there has been emerging interest in harnessing these concepts in clinical care by testing the efficacy and safety of higher-intensity exercise protocols in postmenopausal women with osteopenia/osteoporosis [22–25]. Such programs were traditionally under-studied due to concerns of tolerability and precipitating fractures in this high-risk population. In a meta-analysis, Kistler-Fischbacher et al. identified only a small proportion of exercise trials (4/63; < 10%) that incorporated high intensity protocols in studies in postmenopausal women [8]. High intensity was defined as loads greater than 80% 1RM (one repetition maximum) at frequency of < 8 repetitions for resistance training and ground reaction forces > 4 × bodyweight for impact training. High-intensity resistance interventions, particularly when combined with impact training, were shown to be most effective for increasing lumbar spine (LS) BMD, although only one-quarter of studies were in women with osteopenia/osteoporosis. Other meta-analyses in this field have consistently shown that LS BMD gains (~ 2–3%) can be achieved with high-intensity exercise protocols, however evidence is often of low-certainty due to considerable heterogeneity [26, 27]. This limitation in collating existing data may be partly explained by heterogeneity amongst moderate-intensity exercise protocols and variation between analyses in how ‘high-intensity’ is defined [8]. Certainly, LS BMD gains were not seen in a meta-analysis of moderate-to-low intensity progressive resistance exercise trials in mostly postmenopausal women [28]. Unsurprisingly, low-impact exercises (walking), and non-weightbearing exercises (cycling, swimming) do not exert a positive effect on BMD [29, 30].

#### Emerging Clinical Trial Evidence Supports Greater Osteogenic Effect at the Lumbar Spine with High-intensity Training

Exercise frequency of at least two sessions per week have been shown to positively influence LS BMD response, particularly if the duration of the program exceeds 12-months [31]. However, hip BMD has consistently been shown to be stable, at best, in response to even high-intensity resistance training. In an RCT in predominantly older women (n = 162, mean age 67-years) who were osteopenic or at high falls risk (Osteocise), a 12-month multimodal exercise program incorporating high-velocity progressive resistance training, moderate-impact weightbearing exercise and highly challenging balance exercises resulted in modest net BMD gains of 1.0–1.1% at the LS and femoral neck (FN), with no change at the total hip (TH) [22]. A greater net bone density response at the LS (+ 2–4%) and maintenance effect compared to low-intensity exercise at FN BMD (+ 0.3% vs −2.0%, net difference 2.3%, *p* = 0.025) were demonstrated with an 8-month high intensity resistance and impact training (HiRIT) program studied in postmenopausal women with mild-moderately low bone mass in two separate RCTs (LIFTMOR, MEDEX-OP, n = 106 total randomised to exercise intervention) [23, 24]. This HiRIT program incorporated resistance exercises conducted in weightbearing positions and targeting muscles in the mid-lower back including deadlifts, back squats and overhead presses, and high-impact jump exercises (landing after dropping from a chin-up bar) [32]. HiRIT was well-tolerated with average adherence > 80% [16, 17]. Maximal weight lifted during deadlift was significantly positively associated with LS BMD response (MEDEX-OP), supporting a dose–response relationship between skeletal load magnitude and BMD gains [17]. A 13-month trial in early postmenopausal women with osteoporosis/osteopenia similarly found a significant BMD effect at the LS but not the TH with a high-impact, high-intensity resistance program [25].

In LIFTMOR and MEDEX-OP, HiRIT was associated with structural changes in the proximal femur using 3D-modelling software to conduct post-hoc analyses of hip DXA scans [33], including improvements in FN cortical thickness, and TH trabecular volumetric BMD [23, 34]. Hence, the proximal femur may adapt to loading by altering its morphology (rather than bone density) and so the complete picture of benefits of skeletal loading at the hip (structure, geometry) may not be fully captured by DXA (areal BMD) [23, 34, 35]. This may be of substantial clinical relevance as cortical bone parameters such as thickness are major contributors to femoral neck strength and fracture-resistance [36, 37]. However values representing least significant change are not reported for these 3D hip DXA parameters so conclusions from this data should be extrapolated with caution. In Osteocise, there was no benefit of multimodal exercise on distal femoral or proximal tibial trabecular bone microarchitecture (TbM) [38], whilst a meta-analysis indicated limited evidence to assess effects of exercise on TbM at the distal tibia and radius in postmenopausal women potentially due to insufficient power [39].

#### Uncertainties Around Maintenance of Bone Density Gains with Sustained Exercise and Loss of Bone Density Gains During De-training

Data is scarce investigating persistence of osteogenic effects over sustained long-term participation in an exercise intervention. In a 6-month real-world extension of a 12-month RCT (Osteocise), there was persistence of net femoral neck BMD gains (~ 2%) despite a slight decline in mean exercise adherence to < 50%, but lumbar spine BMD effect was lost [32]. It is uncertain whether progressive BMD gains can be achieved with continuous incremental skeletal loading, or at what timepoint a plateau in osteogenic response may occur. The previous study suggests a lower maintenance frequency of exercise may be able to maintain the initial benefits achieved with skeletal mechano-adaptation [38]. However, a minimum threshold of exercise frequency and/or intensity to maintain BMD gains is not established and would be of great relevance particularly in older populations who may find it challenging and/or cost-prohibitive to maintain an intensive supervised training program. Prior studies exploring this in postmenopausal women have been conflicting, limited by imbalance in long-term attrition rate, lack of randomisation to exercise groups and poor exercise adherence [40–42]. An abstract explored whether BMD gains after 8-months of HiRIT were maintained during a 3-year extension of the LIFTMOR trial which included ~ 50% of initial participants [43]. Those who continued HiRIT after the trial exhibited significant ongoing improvement in BMD at the LS (8.63 ± 5.29% vs 2.18 ± 5.65%, *p* = 0.042) and FN (3.67 ± 4.45% vs 2.85 ± 5.79%, *p* = 0.014) compared to those who did not. Another unanswered question is whether, and how rapidly, interruption or cessation of training results in loss of BMD gains. Few small studies in postmenopausal women showed BMD can return to baseline levels or approach that of controls after 12-months of detraining, although earlier follow-up has not been investigated [44–46].

### Effects of Exercise on Risk of Fragility Fractures in Postmenopausal Women

#### Limitations in Existing Data Supporting Fracture Risk Reduction with Exercise

It is plausible that exercise may also reduce fragility fracture risk, particularly when combining i) balance/functional exercises to address falls prevention with ii) resistance/impact training to promote bone strength. However, there is no definitive evidence that any specific exercise program can reduce fracture risk, as no RCT has been sufficiently powered to demonstrate this outcome. This is likely due to difficulty testing an exercise program consistently across a sufficiently large population over adequate duration to show a difference in fracture rates. Meta-analyses of trials in community-dwelling older adults (predominantly women) have indicated exercise interventions can reduce falls-related fractures and major osteoporotic fractures by ~ 20–30% compared with control groups [47–51]. These trials mostly focussed on balance and functional exercises alone or in combination with resistance exercise. However, evidence-based recommendations for any specific type or characteristics of an exercise program for optimal fracture risk reduction are limited as subgroup analyses have not demonstrated significant fracture prevention with any single mode of training [47, 48]. Supervised exercise may exert a greater effect on fracture risk reduction, however longer duration > 12-months and progressive intensity have not been shown to influence fracture outcomes [48–51]. Data are limited as only few studies have included fractures as the primary outcome, vertebral fractures have been less frequently studied, intensity of training is often insufficient or not specified, and methodological heterogeneity between trials may dilute the fracture risk-lowering effect of more robust exercise programs [48, 49].

#### Safety Considerations of HiRIT

It is possible that high-intensity exercise, particularly in postmenopausal osteoporotic women at high risk of fracture, may place women at risk of injuries, falls or fractures related to the exercise intervention itself. In postmenopausal women at elevated risk of fragility fracture, concerns around loading through the thoracolumbar spine during resistance and impact training historically hampered the investigation of these exercise strategies at a sufficient intensity to generate an osteogenic response in this priority population [8]. However, more recent RCTs in postmenopausal women with low bone mass have demonstrated this to be safe and well-tolerated [22–25]. Important safety principles were followed in these trials included adequate supervision by an exercise physiologist or physiotherapist, individualisation, initial familiarisation periods to ensure correct lifting technique and progressively graded intensity [23, 24], principles which should be actively applied in any clinical setting. Suitable HiRIT participants should be able to mobilise independently and be able to understand and follow the instructions during their supervised sessions to optimise safety. The LIFTMOR trial (in which ~ 1/3 participants had prior vertebral fracture) conducted morphometric assessment for incident vertebral deformity using lateral thoracolumbar spine DXA scans. Reassuringly, exposure to an 8-month HiRIT program did not precipitate any signs of harm such as increased thoracic kyphosis, new or progressive vertebral fracture or worsening scoliosis [52]. Participants of HiRIT program also reported higher levels of physical activity enjoyment compared to those receiving low intensity unsupervised home-based exercise (Kruskal–Wallis H, χ2(2) = 10.958, p < 0.001), with a mean rank physical activity enjoyment score of 34.6 for control and 52.4 for HiRIT (unpublished data).

#### Is HiRIT Safe in Individuals with Recent Fragility Fracture?

There is, however, a lack of evidence on safety of such high-intensity exercise programs in patients who have sustained a recent vertebral fracture (< 12 months), as such patients were excluded from the aforementioned trials [22–25]. A Cochrane systematic review in 2019 found insufficient evidence to explore effects of exercise in patients with any history of vertebral fracture on risk of incident fracture, falls or adverse events, with analyses limited by sample size and diversity in exercise interventions and follow-up duration [53]. A more recent meta-analysis assessed outcomes specifically of progressive resistance and balance training in mostly postmenopausal women with prior osteoporotic vertebral fracture (n = 1289). Although not reporting on bone outcomes, this meta-analysis did show improvements in quality of life, pain, kyphosis, dynamic mobility, functional reach and fear of falling [54]. Exercise intensity was not reported and subgroup analyses based on age and duration of exercise intervention were tentative due to limited statistical power. Recency of vertebral fracture was not specified in either meta-analysis [53, 54]. Back-strengthening or general rehabilitative exercise after vertebroplasty or kyphoplasty for osteoporotic vertebral fracture have also been shown to improve pain and self-perceived disability at 6–12 months without any increase in refracture risk compared with non-exercising controls [55]. However, comparisons regarding cement leakage, an important adverse event of this procedure, were unable to be assessed.

A Cochrane systematic review in 2022 and two other recent meta-analyses support the use of balance and functional or progressive resistance exercises after hip fracture surgery in older adults (predominantly women > 80 years old) for benefits in dynamic mobility and walking speed, although with low certainty of evidence due to substantial heterogeneity between exercise protocols [56–58].

### Proposed Mechanisms for Osteogenic Effects of Exercise and New Insights

#### Mechanosensitive Bone Tissue, the Osteocyte Canalicular-lacunar Network and Wnt Signaling

**Fig. 1:**
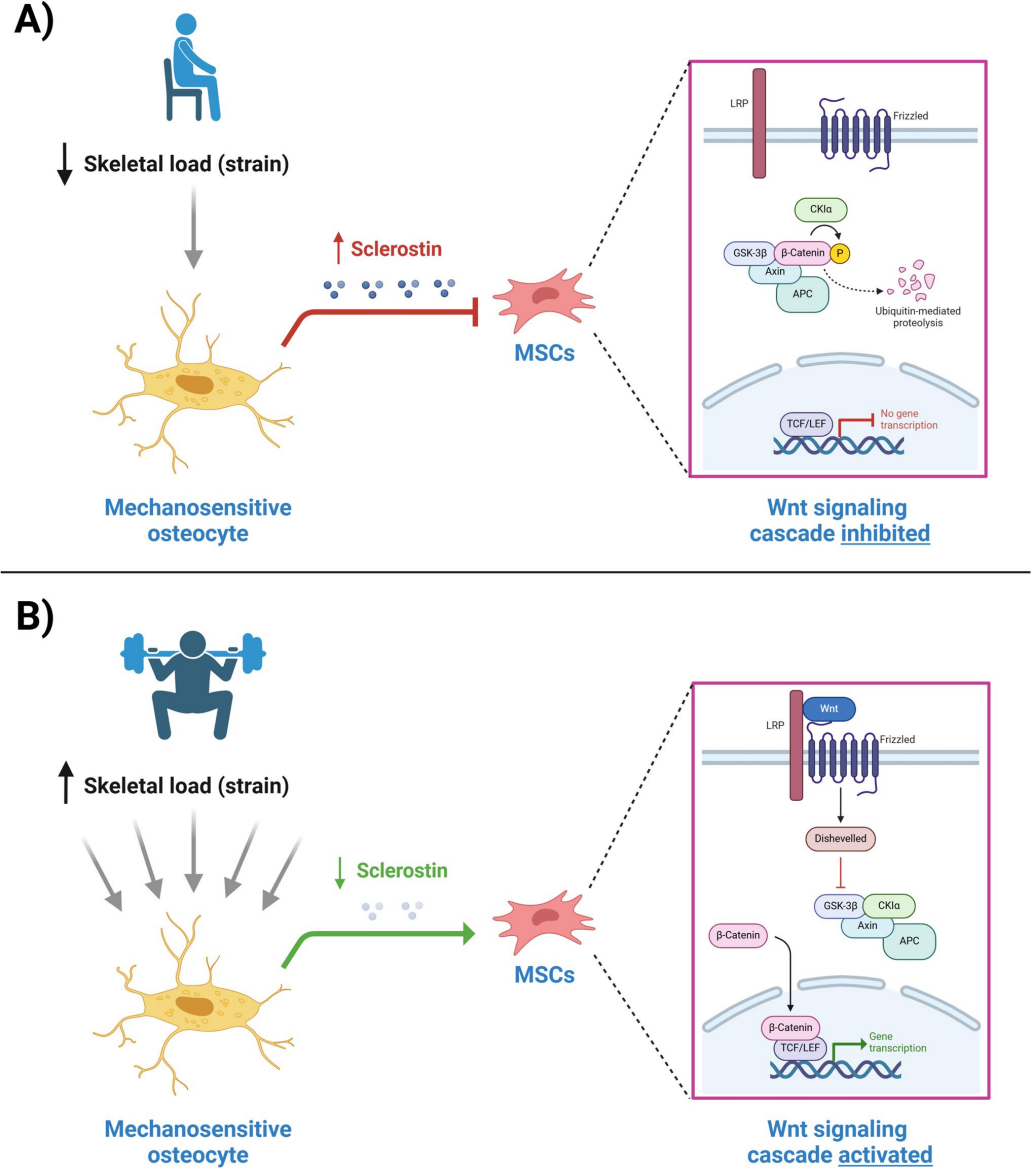
Osteocytic changes in sclerostin expression mediate bone mechanotransduction to skeletal loading. MSC = mesenchymal stem/stromal cell; LRP = low-density lipoprotein receptor-related protein; GSK-3β = glycogen synthase kinase-3 beta; CKIα = casein kinase 1 alpha; APC = adenomatous polyposis coli; TCF/LEF = T cell factor/lymphoid enhancer factor family of transcription factors. Figure created with BioRender.com. Figure 2A represents a skeletally ‘unloaded’ model, for example during sedentary activity. Minimal strain is detected by the mechanosensitive osteocytes resulting in upregulation of *SOST* expression. Increased sclerostin inhibits the canonical Wnt signalling pathway in mesenchymal stem/stromal cells by preventing binding of Wnt proteins to the LRP/frizzled receptor complex, ultimately resulting in ubiquitin-mediated proteolysis of cytosolic β-catenin and hence inhibition of β-catenin mediated changes in osteogenic gene expression. Figure 2B represents a skeletally ‘loaded’ model, for example during HiRIT. Increased strain is detected by the mechanosensitive osteocytes resulting in downregulation of *SOST* expression. Reduced sclerostin allows Wnt to active LRP/frizzled receptor complex, ultimately resulting in protection of β-catenin against proteolysis, β-catenin nuclear translocation and modulation of transcription factors involved in gene expression promoting osteogenic differentiation

Bone tissue harbours the capacity for *mechanotransduction*; converting mechanical stimuli (i.e. skeletal load) into biochemical changes in cell biology and signalling (i.e. bone remodelling, modelling) [59]. These processes facilitate skeletal adaptations in response to load. Put simply, progressive loading of the skeleton (from weightbearing, or skeletal muscle contraction) at magnitudes above that experienced during habitual daily activity will induce an adaptive local osteogenic effect to withstand the increased load. The Wnt signaling pathway drives osteoblastogenesis and has been identified as a crucial pathway in mediating mechanotransduction (Fig. 1). Loading results in downregulation of *SOST* expression (which encodes sclerostin, a negative regulator of Wnt signaling) and hence enhances bone formation [59]. Osteocytes, the most abundant cell in bone, are housed in an intricate lacunar-canalicular network and are considered the ‘*mechanosensors’* in this model [60]. However, the mechanisms whereby osteocytes sense mechanical load and trigger alterations in bone metabolism are not fully established. The leading hypothesis is that load-induced shifts in canalicular extracellular fluid result in flow-induced shear stress across the osteocyte surface, which are sensed by mechanosensitive channels and may trigger mediators such as voltage-sensitive calcium influx and/or paracrine release of messengers such as prostaglandin E2 and nitric oxide [60–62].

Regardless of the underlying mechanisms, the importance of Wnt signalling and sclerostin downregulation in mediating the osteogenic effects of skeletal loading have been established [63–66]. However, aging bone in mice is associated with reduced integrity of the osteocyte lacunar-canalicular network [60]. Whether this translates to blunted mechanotransduction in older postmenopausal women is of considerable clinical relevance, although HiRIT trials appear reassuring against this [23, 24]. Exercise-induced changes in serum sclerostin concentrations and bone turnover markers may shed further light, however data are scarce. Two small uncontrolled studies in postmenopausal osteopenic women or older adults found conflicting evidence on resistance/impact exercise effect on bone turnover markers (P1NP, CTx) whether measured acutely post-session or at the end of a 3-month program [67, 68]. In an RCT, physically active predominantly older women undergoing a home-based strengthening exercise program (30 min, 3x/week) had a marginal annual decrease in sclerostin levels over 3-years compared to those undergoing control exercise [69]. Another study showed no change in serum sclerostin, or bone turnover markers 24-h after a bout of jumping exercises in postmenopausal vs premenopausal women [70].

#### Muscle/bone Tissue Crosstalk, Myokines and Small Extracellular Vesicles

**Fig. 2:**
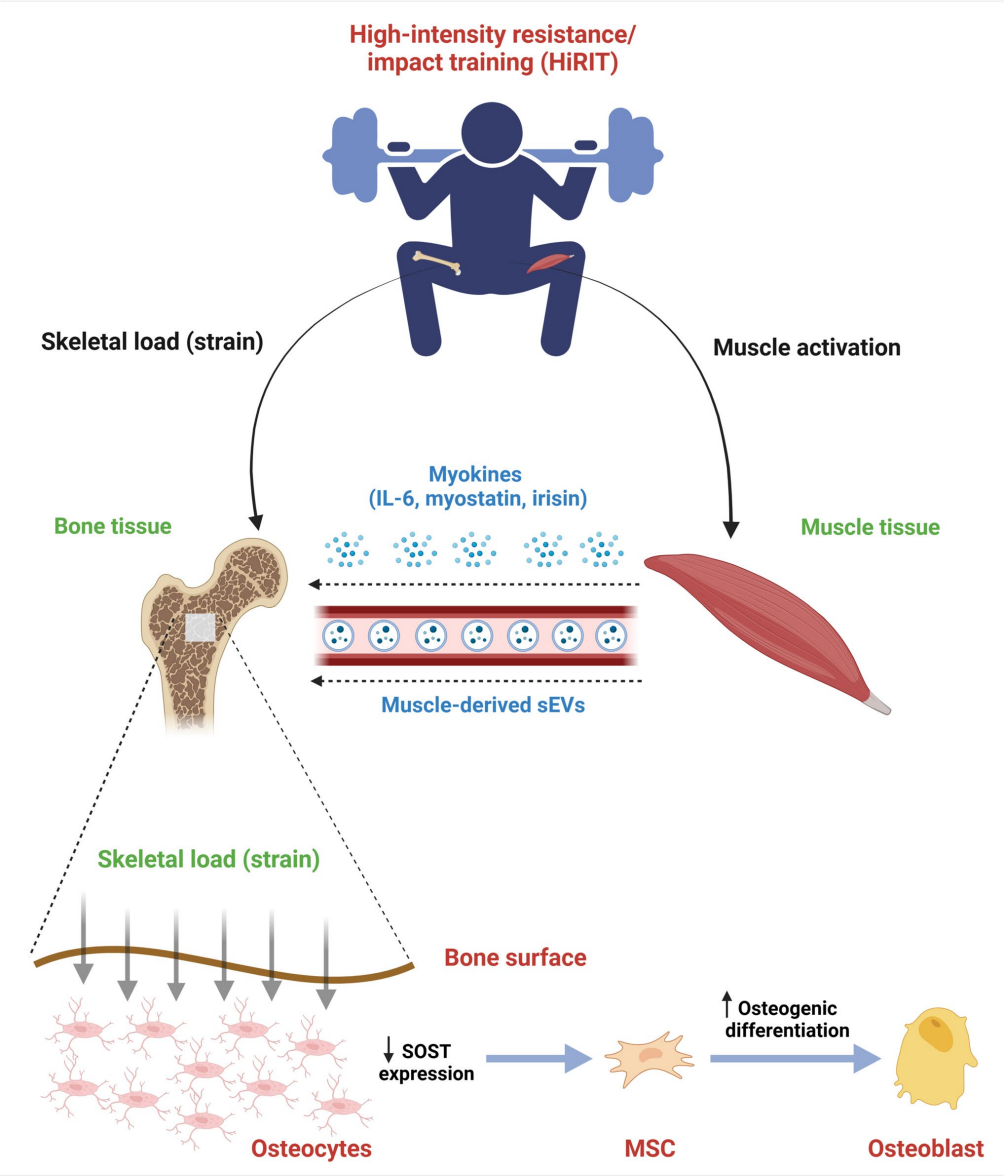
Conceptual representation of potential mechanisms for osteogenic effects of exercise. HiRIT = high-intensity resistance and impact training; IL-6 = interleukin-6; sEVs = small extracellular vesicles; SOST = sclerostin gene; MSC = mesenchymal stem/stromal cell. Figure created with BioRender.com. HiRIT in a postmenopausal woman may exert positive effects on bone tissue through various musculoskeletal pathways. The increased skeletal load (strain) is detected by mechanosensitive osteocytes embedded in the bone matrix. Osteocytes then downregulate *SOST* expression which results in increased osteogenic differentiation (mesenchymal stem/stromal cells undergo differentiation into bone-forming osteoblasts). Activation of skeletal muscle tissue may also trigger release of myokines known to modulate bone metabolism (IL-6, irisin, myostatin) or other myokines/mediators travelling in muscle-derived small extracellular vesicles

Other than synergistic roles between bone and muscle in facilitating locomotion, molecular signals which are derived from muscle tissue and act on bone tissue (myokines) may also modulate tissue crosstalk, particularly during mechanical stimulation (i.e. exercise). Muscle and bone are also intimately connected in pathological states of musculoskeletal aging (sarcopenia and osteoporosis) regarding risk factors for and consequences of both conditions. A comprehensive review on these topics can be found elsewhere [71]. Candidate myokines including myostatin, irisin and interleukin-6, have been shown to be released by skeletal muscle during exercise and impact differentiation of critical bone-remodelling cells (osteoblasts, osteoclasts) (Fig. 2) [72]. Studies in humans have shown resistance exercise is capable of inducing changes in circulating concentrations of these myokines, however the majority were conducted in healthy young men, with very limited data in postmenopausal women [73].

Studies on predominantly healthy young men have also demonstrated that acute bouts of endurance/aerobic exercise trigger rapid release of circulating small extracellular vesicles (sEVs), potentially acting as mediators of crosstalk between muscle and other target tissues [74, 75] (Fig. 2). Could some degree of skeletal adaptation to exercise be induced by myokines transported by muscle-derived EVs to target sites in bone tissue? In vitro, muscle-derived EVs have been shown to influence bone remodelling by acting on osteoblast and osteoclast precursor cells, and in-vivo, were demonstrated to reverse a model of disuse osteoporosis [76, 77]. However, the majority of studies investigating exercise-associated release of sEVs focus on acute bouts of cycling, running and aerobic exercise in men, which is of less relevance to the response of bone-targeted exercise in postmenopausal women with low bone mass. There is very limited data on resistance exercise effects on circulating sEVs [78] and to-date, no studies have explored this in a postmenopausal cohort.

### Can we Raise the Bar by Combining Bone-targeted Exercise with Osteoporosis Medication for Optimal Outcomes in Postmenopausal Bone Health?

#### Limited Evidence Suggests no Added Benefit of Combining Antiresorptives with Resistance and Impact Exercise

Broadly speaking, an interdisciplinary treatment approach that includes exercise plus osteoporosis medication might be expected to exert multi-dimensional benefits on fracture risk reduction and musculoskeletal health by increasing bone mass, enhancing bone quality, and reducing falls risk. Clinical practice guidelines generally recommend exercise as part of holistic osteoporosis management, primarily using progressive resistance exercise with the incorporation of challenging balance exercises to target falls prevention [79, 80]. However, very few studies have assessed whether exercise can modulate the osteogenic effects of osteoporosis pharmacotherapy. Results from three RCTs published over two decades ago did not show any additional benefit on BMD when adding 6–12 months of either moderate-intensity resistance or impact training alone to oral bisphosphonates (alendronate, etidronate) [81–83]. However, these studies were predominantly in healthy postmenopausal women who did not necessarily have low BMD which may have hampered the effect. Further, the exercise interventions were likely sub-optimal and did not exert sufficient osteogenic stimulus to improve BMD. The DO-HEALTH study combined vitamin D supplementation with home-based exercise in healthy older adults and similarly did not demonstrate any BMD benefit over 3-years. However, this study included a low-intensity exercise intervention without progressive resistance, i.e. with insufficient osteogenic load stimulus [84]. MEDEX-OP assessed HiRIT in postmenopausal women (BMD T-score < −1.0) and stratified randomisation by presence of established antiresorptive therapy, mostly consisting of denosumab [24]. Subgroup analyses did not demonstrate additional benefit of exercise above antiresorptive therapy although were likely under-powered due to under-recruitment of participants on established pharmacotherapy. A prospective controlled study simultaneously commencing participants on specific osteoporosis pharmacotherapy and bone-targeted exercise would likely provide a more definitive answer and address the heterogeneity in type and duration of medication exposure.

#### Osteoanabolic Pharmacotherapy and Exercise in Combination: Future Directions

Twelve months of treatment with romosozumab (anti-sclerostin monoclonal antibody) has demonstrated potent osteoanabolic properties and robust fragility fracture reduction against placebo and an oral bisphosphonate, alendronate [85, 86]. Given the importance of *SOST* downregulation in mediating mechano-adaptive osteogenic responses to skeletal loading, the combination of anti-sclerostin treatment (romosozumab) with bone-targeted exercise may have the potential to harness additive benefits on bone strength by co-modulating osteoblastic signalling pathways. This hypothesis is supported by several murine studies, whereby tibial loading induced a greater local osteogenic response (based on BMD, bone volume on micro-CT and bone formation rate on histomorphometry) in the presence of sclerostin deficiency (either chronic via *SOST* knockout, or acute via anti-sclerostin antibody administration) [87–91]. This animal data is promising, yet there is no published data regarding whether exercise may have additive benefits beyond potent osteoanabolic therapy in humans. Further, studies in mice suggest additive osteoanabolic effects of skeletal loading above sclerostin deficiency may be age-, sex- and bone compartment-specific [87–91]. This may have implications on whether combined approaches using resistance exercise and romosozumab would in fact be additive in a priority population of postmenopausal women with low bone mass. A retrospective study in predominantly postmenopausal treatment-naïve patients (n = 47), presented as an abstract at ASBMR 2024, provides the first indication that greater exposure to physical activity (measured by retrospective questionnaire) during one-year of romosozumab may be associated with greater BMD response at the spine and hip [92]. Those who could ambulate independently had a greater LS BMD response (+ 14% vs −3%, *p* = 0.021) while only a trend was seen in those engaging in sports (+ 15% vs 2%, p = 0.094). Although retrospective and limited by statistical power, this study provides early evidence that skeletal loading and romosozumab may exert additive effects on enhancing BMD. Our group is currently testing this hypothesis prospectively in an 8-month randomised placebo-controlled trial in postmenopausal women with osteoporosis/osteopenia (ROLEX-DUO) [93]. This RCT will explore the effect of romosozumab on lumbar spine, total hip and femoral neck BMD in combination with either high-intensity resistance and impact training or low-intensity exercise, with results anticipated in late 2026.

#### Resistance Exercise to Prevent Bone and Muscle Loss During Effective Weight-loss Therapies

Effective weight loss strategies continue to emerge as a major global scientific priority to combat rising obesity rates, which are highly prevalent in postmenopausal women [94]. However, there are growing concerns over concurrent losses of lean muscle and bone mass, and subsequent implications on musculoskeletal health. Meta-analyses have reported that lean muscle mass accounts for up to 50% of total weight loss when using various weight loss strategies (e.g. caloric restriction, bariatric surgery, pharmacotherapy) [95, 96]. Newer incretin-based pharmacotherapies can achieve dramatic weight loss of 15–25% over a ~ 1-year period [97] and recent RCTs in obese adults and postmenopausal women with osteopenia also indicate modest BMD loss at the spine and total hip with these treatments [98, 99]. The benefits of resistance exercise are established on increasing muscle mass, strength and physical function in healthy adults and older adults with sarcopenia [100, 101] as well as positive effects on bone density. Hence, resistance exercise may also have a role in preserving muscle and bone mass during accelerated weight loss therapies. A secondary analysis of a one-year RCT of mostly female obese adults (n = 195, 64% female) showed that adding high-intensity resistance and aerobic exercise preserved bone mass during liraglutide treatment [98]. The BEACON trial is a 12-month RCT currently recruiting obese osteopenic adults and testing whether resistance exercise plus alendronate can preserve BMD, bone microarchitecture (HR-pQCT) and lean muscle mass better than either alone during dietary weight loss intervention [102].

### Conclusions

Collated clinical trial evidence highlights the importance of challenging balance exercises for falls prevention. High-intensity resistance and impact training, traditionally avoided due to safety concerns in postmenopausal osteopenia/osteoporosis, has recently been demonstrated to be a safe strategy capable of inducing a considerable osteogenic stimulus. Despite the plethora of RCTs investigating exercise for bone health, there remain several unanswered questions, including maintenance of osteogenic responses to skeletal loading and how best to sustain this benefit long-term. A greater understanding of the mechanisms underpinning skeletal adaptations to exercise may open novel strategies to uncover the full potential of exercise on positive skeletal health outcomes. With greater understaning of the osteogenic effects of high-intensity exercise and the development of potent osteoporosis pharmacotherapies, the future presents new opportunities for holistic, cross-disciplinary approaches to the treatment of osteoporosis in an aging at-risk population.

## Key References

- Kistler-Fischbacher M, Weeks BK, Beck BR. The effect of exercise intensity on bone in postmenopausal women (part 2): a meta-analysis. Bone. 2021;143:115697. 10.1016/j.bone.2020.115697.
  - Meta-analysis which demonstrated high-intensity exercise is more efficacious for increasing lumbar spine BMD in postmenopausal women than low-to-moderate intensity exercise This meta-analysis also highlighted the relative lack of trials incorporating high-intensity exercise regimens in this at-risk population.
- Sherrington C, Fairhall N, Kwok W, et al. Evidence on physical activity and falls prevention for people aged 65 + years: systematic review to inform the WHO guidelines on physical activity and sedentary behaviour. Int J Behav Nutr Phys Act. 2020;17:144. 10.1186/s12966-020-01041-3.
  - Large updated systematic review (*n* = 25,160 participants ≥ 60-years, 74% female) showed that falls rates are reduced most with balance/functional exercises (24%) and multimodal exercise incorporating balance/functional exercises plus resistance exercise (28%).
- Watson SL, Weeks BK, Weis LJ, Harding AT, Horan SA, Beck BR. High-intensity resistance and impact training improves bone mineral density and physical function in postmenopausal women with osteopenia and osteoporosis: the LIFTMOR randomized controlled trial. J Bone Miner Res. 2018;33(2):211–220. 10.1002/jbmr.3284.
  - This RCT (*n* = 101) demonstrated that 8-months of high intensity resistance and impact training (HiRIT) improved lumbar spine BMD by ~ 4% compared to low-intensity exercise in postmenopausal women with low BMD. HiRIT was well-tolerated with average adherence > 90%.
- Kistler-Fischbacher M, Yong JS, Weeks BK, Beck BR. A comparison of bone-targeted exercise with and without antiresorptive bone medication to reduce indices of fracture risk in postmenopausal women with low bone mass: the MEDEX-OP randomized controlled trial. J Bone Miner Res. 2021;36(9):1680–1693. 10.1002/jbmr.4334.
  - This RCT (*n* = 115) demonstrated 8-months of HiRIT improved lumbar spine BMD by ~ 2.5% compared to low-intensity exercise in postmenopausal women with low BMD. Subgroup analyses, which were likely underpowered, did not demonstrate any additional benefit of HiRIT above established antiresorptive pharmacotherapy.
- Hettchen M, von Stengel S, Kohl M, et al. Changes in menopausal risk factors in early postmenopausal osteopenic women after 13 months of high-intensity exercise: the randomized controlled ACTLIFE-RCT. Clin Interv Aging. 2021;16:83–96. 10.2147/CIA.S283177.
  - This 13-month RCT (*n* = 54) of early postmenopausal women with low BMD showed a significant BMD effect at the lumbar spine with a high-impact weight-bearing high-intensity resistance training program compared to low-intensity exercise controls.
- Daly RM, Gianoudis J, Kersh ME, et al. Effects of a 12-month supervised, community-based, multimodal exercise program followed by a 6-month research-to-practice transition on bone mineral density, trabecular microarchitecture, and physical function in older adults: a randomized controlled trial. J Bone Miner Res. 2020;35(3):419–429. 10.1002/jbmr.3865.
  - In this RCT of predominantly older women (*n* = 162) with osteopenia or high falls risk, a 12-month multimodal exercise program (high-velocity progressive resistance, moderate-impact weightbearing and highly challenging balance exercises) resulted in modest net BMD gains of 1.0–1.1% at lumbar spine and femoral neck. A 6-month extension suggested maintenance of BMD effect in those who continued the exercise despite reduced frequency.
- Watson SL, Weeks BK, Weis LJ, Harding AT, Horan SA, Beck BR. High-intensity exercise did not cause vertebral fractures and improves thoracic kyphosis in postmenopausal women with low to very low bone mass: the LIFTMOR trial. Osteoporos Int. 2019;30(5):957–964. 10.1007/s00198-018-04829-z.
  - In this secondary safety analysis of LIFTMOR trial, 8-months supervised HiRIT in postmenopausal women with low BMD did not result in any new/worsening vertebral fractures. The HIRIT program delivery ensured certain safety principles were maintained including supervision, individualisation and progressive intensity increments.

## Data Availability

No datasets were generated or analysed during the current study.
